# Improving Adherence to Post‐Cervical Biopsy Sexual Abstinence in Kenyan Female Sex Workers

**DOI:** 10.1111/aji.12520

**Published:** 2016-05-24

**Authors:** Julie Lajoie, Genevieve Boily‐Larouche, Kelsie Doering, Juliana Cheruiyot, Julius Oyugi, Kristina Broliden, Joshua Kimani, Keith R. Fowke

**Affiliations:** ^1^ Department of Medical MicrobiologyUniversity of ManitobaWinnipegMBCanada; ^2^ Department of Community Health SciencesUniversity of ManitobaWinnipegMBCanada; ^3^ Department of Medical MicrobiologyUniversity of NairobiNairobiKenya; ^4^ Kenya AIDS Control ProgramNairobiKenya; ^5^ Department of Medicine SolnaCenter for Molecular MedicineClinic of Infectious DiseasesKarolinska University HospitalKarolinska InstitutetStockholmSweden

**Keywords:** Female genital tract, HIV, immunology, mucosal, prostate‐specific antigens, safety

## Abstract

**Problem:**

Cervical biopsies offer a unique opportunity for studying local immune response. To investigate hormonally induced immune fluctuations in cervical tissues of Kenyan female sex workers, we improved biopsy sampling protocol safety. Here, we report on steps taken to minimize exposure to HIV following two cervical biopsies.

**Methods of study:**

Women were asked to abstain from vaginal intercourse to limit HIV exposure during wound healing with financial compensation. A comprehension tool for informed consent, on‐site detection of prostate‐specific antigens indicating unprotected intercourse within 48 hr, and bi‐weekly text message reminders were implemented.

**Results:**

The implemented methods improved compliance with post‐procedure abstinence by two times (*P* = 0.013). Fourteen days following a cervical biopsy, no sign of genital inflammation or change in HIV T‐cell target proportion were observed.

**Conclusions:**

This study provides new tools for limiting HIV exposure in studies requiring biopsy sampling among women at risk of acquiring HIV.

## Introduction

Worldwide, the majority of HIV transmissions occur through unprotected sexual intercourse.^1^ Given the importance of mucosal transmission, HIV preventive biomedical research needs to focus on the study of protective immune responses at mucosal sites. Unfortunately, the majority of large‐scale HIV vaccine trials conducted recently did not include extensive mucosal sampling. Therefore, the immune responses elicited by vaccines only included systemic responses and not the response elicited at the mucosal portal of virus exposure. Characterizing the systemic response might not be sufficient to understand the nature of the immune protection at the mucosal compartment.^2^, ^3^

Few studies have explored the female genital tract (FGT) immune response. The complex logistics of performing vaginal sampling and the difficulties associated with their analysis have impeded this area of research.^4^ The collection of FGT samples can be achieved with different methods. Cytobrush/scrapper and cervicovaginal lavage (CVL) are among the most common and less invasive methods. CVL collection allows the study of immune proteins, but is not suitable for studying cellular populations.^5^ Cytobrush sampling is a noninvasive technique that collects endocervical mononuclear cells enabling the characterization of cervical immune populations.^6^ However, cytobrush sampling leads to a small yield limiting the number of possible analyses.

Cervical biopsies are another important tool for studying FGT immune response. Collection of small cervical biopsies from the superior portion of the ectocervix with a biopsy forceps is a standardized procedure in routine gynecology for assessment of cervical abnormalities. The information gathered by studying the *in situ* cellular structure is extremely valuable to understand the important juxtaposition of HIV target cells in the tissue as well as epithelial structures and extracellular matrix components. However, cervical biopsies are more invasive than cytobrush sampling techniques, theoretically increasing the risk of acquiring HIV during the healing period.

Understanding the architecture of the genital mucosa and the juxtaposition of HIV targets therein as well as the nature of the factors affecting HIV targets recruitment and mucosal immune response at the portal of entry remain crucial for the development of effective vaccines and other HIV prevention tools. Yet, this information also needs to be generated in most‐at‐risk populations such as female sex workers (FSWs) to ensure the adequacy of the developed interventions for this population. To reduce the risk of HIV acquisition after a cervical biopsy, women are asked to abstain from vaginal intercourse for a defined time period. However, collecting cervical biopsies in FSWs must consider the relatively high HIV exposure risk associated with sex work and the financial implications behind refraining from sexual activities. A previous study conducted in Nairobi, Kenya, showed that collecting cervical biopsies were accepted and well tolerated by FSWs who complied with the 2‐week healing period of sexual abstention.^7^

To understand the impact of sexual hormones on the FGT environment of FSWs from Nairobi, we developed a study protocol where biopsy sampling was required. It was estimated by the study team that biopsy sampling would be the most accurate way of obtaining information about the impact of hormonal factors on mucosal immune response and epithelial structures.

Here, we report on how, within a group of FSWs, new measures improved the compliance of sexual abstinence following a cervical biopsy, which would result in reduced potential HIV exposure and improved safety for research participants.

## Materials and methods

### Study Population

**Figure 1:**
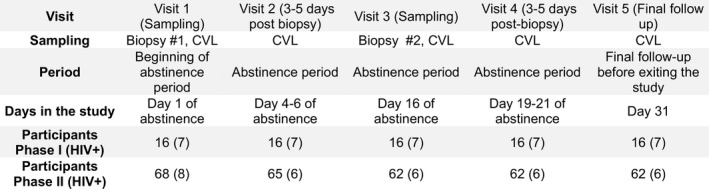
Schematic representation of the study visits. Participants were followed for 5 visits (1 month). The first biopsy sample was collected at visit 1, and a follow‐up visit to monitor the healing process took place 3–5 days post‐procedure (visit 2). The second biopsy (visit 3) was taken 2 weeks later, and healing was monitored 3–5 days later (visit 4). After the 31 days of sexual abstinence, participants came to the clinic for a final exam of the biopsy sites and a final follow‐up (visit 5).

We recruited 84 premenopausal FSWs who were not on hormonal contraceptive from the Pumwani Sex Worker Cohort in Nairobi, Kenya. Seventy‐eight participants completed all the study visits (Fig. 1). This study was conducted in two phases. We first enrolled 16 participants during Phase I (May‐July 2013) and 68 during Phase II (February‐August 2014). In Phase I, 16 of 16 participants completed the study. In Phase II, 68 participants underwent the first biopsy and 62 completed all the study visits (Fig. 1). For both phases, the inclusion criteria were as follows: (i) being active in sex work; (ii) uterus and cervix present; (iii) 18 to 50 years of age, while being premenopausal; (iv) willing to undergo cervical biopsies and undertake a month‐long period of sexual abstinence; (v) for HIV‐infected women, a CD4 count over 350/mm^3^ and antiretroviral therapy naive. Urine was collected at visit 1, 3, and 5, and *N. gonorrhoea* or *C. trachomatis* were diagnosed using Roche Amplicor kits. Syphilis was diagnosed serologically during the same visits, and a Gram stain from vaginal swabs was performed to determine Nugent Scoring for the presence of bacterial vaginosis infection. All participants were screened for HIV using rapid test (Determine, Inverness Medical, Shinjuku‐ku, Japan), and positive tests were confirmed by ELISA (Vironostika, Biomerieux, Marcy l'Etoile, France). As the purpose of the overall study was to study the mucosal tract of sex workers, both HIV‐infected and HIV‐uninfected participants were enrolled in the study. Of the total 84 participants, 15 were HIV infected. Participants positive for non‐HIV sexually transmitted infections (STI) at baseline were excluded from the study and treated according to Kenyan treatment protocols. Ethic review boards from the Universities of Nairobi and Manitoba approved the study. Each participant signed a consent form.

### Clinical Follow‐up

During the planning phase of the study design, the team organized a community consultation with the FSW peer leaders to discuss the feasibility of the study and to establish a financial compensation rate that was equivalent to the daily average income for FSW working in the Pumwani region of Nairobi. It was agreed that a daily compensation of 1000kes (~10US) was appropriate. The financial compensation for the loss of income was provided at every biopsy sampling visit. During this meeting, the peer leaders estimated that abstaining from sex work for 1 month was feasible with the support of the financial compensation. The study obtained the full support of the community peer leaders and was initiated a few months later at the Majengo clinic. The clinic is located in the heart of the Pumwani district where most of the recruited participants practice sex work and is part of the Sex Workers Outreach Program (SWOP), a collaborative partnership between the Universities of Manitoba and Nairobi, providing care and services to promote the health, safety, and well‐being of FSWs.

The participants were followed for 1 month and came to the clinic for five visits. Cervical biopsies were obtained by a trained gynecologist at visits 1 and 3. Visit 1 took place a median of 14 days (range 6–43 days) after the recruitment visit, which also included STI screening. Follow‐up visits (visits 2 and 4) took place 3–5 days after the biopsy sampling, and the gynecologist monitored the healing process (Fig. 1). The biopsy site was considered healed when no bleeding from biopsy site, no hyperaemia, and no abnormalities were detected during the macroscopic evaluation. A final follow‐up visit (visit 5) occurred 14 days (range 12–28 days) following the second biopsy. CVL samples were collected at every visit, and cervical cells were collected using endocervical cytobrush and ectocervical spatula scraping. Biopsies were conducted for research purposes only as any cervical abnormalities detected before entering the study would have been referred to a specialist clinic and the woman would have been excluded from entering the study. Indeed, one enrolled participant presented a cervical anomaly (cystocoele) at visit 1 was referred for appropriate care and was discontinued from the study. Overall, five other participants did not complete the study. One participant was diagnosed with a syphilis infection and referred for treatment; one HIV‐infected participant had a CD4 count lower than 350; and three participants were unwilling to follow the post‐procedure directive for sexual abstinence.

The length of the post‐procedure abstinence period was established according to previous observations from Hasselrot et al. showing that healing was mainly fully completed after 2–5 days. However, because the time required to achieve complete healing with no signs of macroscopic hyperaemia could be up to 11 days for some women,^7^ we extended the period of abstinence to 14 days to ensure complete healing for all. When the biopsy wounds were properly healed, the participants were cleared to exit this part of the overall study. If not, they were advised not to resume sex work until healed.

### Pilot Study (Phase I)

#### Counseling

Participants' safety relied on compliance to sex abstention, proper usage of condoms if abstaining was not possible, and efficient monitoring of the healing process. A fulsome understanding of the risks and implications was required to limit the risk of HIV infection or transmission, and the efficacy of the counseling delivered by the study nurse was central in this process. During Phase I, potential participants received explanations about aims, benefits, risks, and implications of the study. Counseling about HIV and condom use was administered by the study nurse. HIV education is part of the routine counseling at the clinic, and the nurses have experience in delivering these messages. Participants were given time to think about their potential enrollment in the study and were asked to come back with any questions they had before enrollment was considered. Once all questions were answered and interest in participating established, consent was sought. Consent was obtained for all participants. A questionnaire asking about contraception history, parturition history, health and medication history, and sociodemographic characteristics including education, country of origin, and marital status was answered at the first visit. At each visit, a clinical and behavioral questionnaire asking about the numbers of clients, regular partners, douching habits, condom usage, and alcohol consumption was answered. Finally, a trained nurse and/or gynecologist educated the participant about the importance of abstaining from vaginal intercourse, or use condoms if abstinence was not possible, for limiting the risk of HIV and STI acquisition and transmission after the procedure.

### Phase II

#### Counseling

In supplement of the counseling and education given during Phase I, a comprehension tool to assess the understanding of the study aims, benefits, risks, and implications was added to the regular process in Phase II. After the counseling session, a card containing the five most important points about the study was given to the participant. The five statements discussed with the nurse were as follows: (i) I will have to come to the clinic every 2 weeks for a duration of 3 months (a total of 6 visits), (ii) I will be tested for the presence of sexually transmitted diseases such as HIV. If I test positive, I will receive free care, (iii) Two cervical biopsies will be performed during the study, (iv) I have the right to stop my participation in the study at any time, (v) Due to increased risk of acquiring HIV after the biopsy, I will have to stop sexual activity for a duration of 2 weeks post‐biopsy (2 biopsies = 1 month of sex break). After reading the tool, the nurse asked the participant to describe their interpretation of these statements. If the participant was illiterate, the nurse assessed the participant understanding by formulating the five points into question format: (i) What is the study about? (ii) How often do you have to come to the clinic? (iii) Is there something special about this study that I will have to do? (iv) Can you have sex during the whole study? (v) What kind of samples will we ask you to give? If the participant answered correctly to all questions, she was considered to properly understand the risk and conditions of the study and the study nurse could proceed with the signature of the consent form. Comprehension was assessed orally and evaluated by the interviewer. When wrong answers were given, the study nurse explained again the implications of the study to the participants until a full understanding was achieved. If understanding was not fully achieved, the participant was not enrolled in the study. Comprehension tool and consent forms were available in English and Swahili. The interview and consent were conducted in the participant's preferred language.

### Short Message Service

In Phase II, a program was implemented to automatically send short message service (SMS) text messages to the participant's cell phone. All participants had access to a cell phone (none were provided by the study) where they could receive private messages. SMSs were sent at the beginning and end of the week as a reminder to abstain from vaginal intercourse during the healing period. A message in Swahili was predetermined by FSW peer leaders during a second community consultation meeting. To ensure privacy, the message was selected to be understood only by the participants and could not be associated with the participant's involvement in sex work or in the study.

### Biopsy Sampling

Two ectocervical biopsies were performed at 2‐week interval. The biopsy was performed using Schubert biopsy forceps (model ER058R; Aesculap, Tuttlingen, Germany) by a trained gynecologist. At each cervical biopsy visit, two punches of 3 mm^2^ from the superior portion of the ectocervix were taken. Ferric subsulfate solution gel (Monsel solution; Gynex, Washington, DC, USA) was applied on the wound to prevent excess bleeding. The biopsy collected provided enough tissue to explore the cellular juxtaposition of HIV target cells. The gynecologist ensured that bleeding was stopped before the participant could leave the clinic. The tissue samples were snap‐frozen in liquid nitrogen and cryopreserved for later assessment.

### Cervicovaginal Lavage and Cervical Cells Collection

Two milliliters of sterile 1× phosphate‐buffered saline was used to wash the ectocervix and collected from the posterior fornix. In Phase I, samples were kept on ice and transferred to the laboratory within 2 hr. The samples were centrifuged and stored at −70°C until batch analyses for PSA detection were performed. In Phase II, the cervicovaginal lavages (CVLs) were collected and a portion immediately used to detect the presence of PSA, with the remaining volume cryopreserved for later use. Cervical cells were collected from an endocervical cytobrush and ectocervical spatula scraping in 5 mL of PBS. Cells were processed according to Juno et al^6^ and stained with various markers to allow for HIV target cell identification (Live‐dead‐PE‐Texas‐Red (Invitrogen, Burlington, ON, Canada), CD3‐PEcy5, CD4‐FITC, CCR5‐V450, CD161‐APC, CD69‐PECy7, HLA‐DR‐APC‐H7, CD8‐V500, (BD Biosciences; Biosciences, Mississauga, ON, Canada). The proportion of HIV target cells among CD4 T cells was determined by flow cytometry. Data were acquired on a BD LSR II cytometer (BD Biosciences) and analyzed with FlowJo Software (version 10.0.7; Tree Star, Ashland, OR, USA).

### Measurement of Cytokine Concentrations in the CVL

Cervical concentrations of the pro‐inflammatory cytokines IL‐8, MIP1β, MIP3α, MIP1α, MCP‐1, MIG, IP‐10, IL‐1α, IL‐1β, and TNF‐α were measured by Milliplex (Millipore, Merck KGaA, Darmstadt, Germany) according to the manufacturer's instructions and analyzed on the Bio‐Plex‐200 (Bio‐Rad, Mississauga, ON, Canada). The cytokines were selected because of their previous association with genital transmission of HIV.^8^, ^9^, ^10^ CVL were incubated overnight with the capture beads to improve the sensitivity of the detection. The protocol was adapted as in Lajoie et al.^9^

### Rapid Prostate‐Specific Antigen Testing

**Figure 2:**
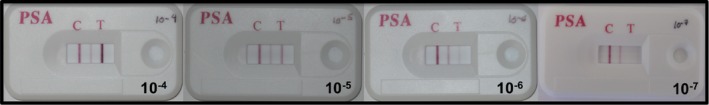
PSA‐semiquant cassette test serial dilutions to indicate PSA concentrations. Semens were diluted in PBS. A total of 120 ul of the semen dilutions were loaded on the immunochromatographic test strip cassette (Seratec PSA Semiquant, Germany). Negative test results were defined by the presence of bands in the control (C) and internal standard columns, but not in the test result column (T). A positive test showed bands in the control (C), the internal standard, and the test result columns (T). PSA concentration >1 ng/mL were detectable by the kit, which corresponded to a 10^−6^ semen dilution as tested by us.

One hundred and twenty microliters of undiluted CVL was loaded directly to the immunochromatographic test strip cassette (Seratec PSA Semiquant, Göttingen, Germany). Results were read after 10 min of incubation at room temperature allowing the sample to migrate through the test strip. Negative test results were defined by the presence of bands in the control (C) and internal standard columns, but not in the test result column (T). A positive test showed bands in the control, the internal standard, and the test result columns. In the absence of an internal standard and/or control bands, the test was considered invalid and the assay was repeated using another strip. The color intensity in the internal standard column represents a concentration of prostate‐specific antigen (PSA) of approximately 4 ng/mL (Fig. 2). Bands were visible at a concentration as low as 1 ng/mL. PSA concentration >1 ng/mL indicated the presence of seminal fluid (PSA) and thus implicates that unprotected sexual intercourse were more likely to have happened within the last 48 hr.^11^ The same method was used for both phases. During Phase I, bulk PSA testings from frozen CVL were performed in the research laboratory, 3 months after the participant's visit. During Phase II, the participant witnessed the PSA testing performed at the clinic on fresh CVL the day of the visit. We validated the assay for CVL sample by comparing semen dilution curves in the kit buffer or a CVL sample negative for PSA. The threshold of detection (10^−6^) was the same for both conditions (data not shown).

### Statistical Analysis

Analyses were performed using SPSS (version 22, IBM Corporation, Armonk, NY, USA). The difference in the proportion of women being positive for at least one PSA test, answering positively to baseline characteristics or showing signs of macroscopic hyperaemia or cervical inflammation (defined by having at least 5/10 pro‐inflammatory cytokines in the 75th percentile^8^) was compared using Fisher's exact test (when the expected count was less than 5) or chi‐square test (when the expected count was greater than 5). Results are presented as percentages with their corresponding *P*‐value. A *P*‐value lower than or equal to 0.05 was considered significant. D'Agostino and Pearson Omnibus and Shapiro–Wilk normality test were used to determine the normality of the distribution. Unpaired *t*‐test was performed when data was normally distributed. Not normally distributed data was compared by Mann–Whitney *U*‐test. The difference in the cervical concentrations of inflammatory mediators and HIV target cells at different visits was calculated using the nonparametric Friedman test for repeated measures. When significant, *post hoc* Dunn's multiple comparisons test was applied to compare between visits.

## Results and Discussion

### Compliance with the Post‐Procedure Directives

In Phase I, rigorous counseling was the strategy prioritized to enforce participant compliance to the post‐biopsy sexual abstinence. This procedure was implemented to facilitate proper healing with no exposure to HIV during the healing period. Despite that intent, three participants reported having unprotected sexual intercourse during the abstinence period. We confirmed these results by testing for the presence of PSA 3 months after the end of the pilot study, once the samples were shipped to Winnipeg. PSA was detected for at least one visit during the sexual abstention period in 10 of 16 participants, indicating a lack of compliance in 63% of the participants (Table 1). Participants with a positive PSA result came back for a follow‐up visit 6 months after the end of the pilot phase, and those who were HIV uninfected at the beginning of the study had remained HIV sero‐negative. In all cases of PSA positivity, participants reported having sex with a regular partner and not a casual client.

**Table 1:** Prostate‐Specific Antigen (PSA)‐Positive Detection in Cervicovaginal Lavages Among Female Sex Workers from Nairobi During the Period of Sexual Abstinence for Post‐Biopsy Healing Purposes; Comparison Between Phase I and Phase II of the Study

Number of participants with PSA+ test	Phase I (*n* = 16)	Phase II (*n* = 62)	*P* value
	% (*n*)	% (*n*)	
Any visit during the healing period	63% (10)	29% (18)	0.013*
Visit 2 (3–5 days post biopsy)	19% (3)	10% (6)	0.365†
Visit 3 (Biopsy 2)	19% (3)	7% (4)	0.148†
Visit 4 (3–5 days post biopsy)	25% (4)	11% (7)	0.212†
Visit 5 (Final follow up)	25% (4)	8% (5)	0.087†

*Chi‐Square ^†^Fischer's Exact Test.

Given the poor adherence to the abstinence period observed in Phase I, concerns were raised in our team about the safety of performing cervical biopsies with 2‐week interval, thus requesting a total abstinence period of 4 weeks in FSWs. To palliate for the lack of compliance, additional strategies were implemented and included the use of the consent comprehension tool to improve participants' knowledge about risks and implications, point of care PSA testing to monitor for compliance during the study, and short bi‐weekly text messaging in between visits as a reminder of the importance of not having sexual intercourse during the healing period. These measures were aimed at increasing adherence to the abstinence period and, therefore, safety for the participants. Both Universities of Manitoba and Nairobi ethic boards approved the modifications to the original protocol. After implementing these new methods in Phase II, improved adherence to the post‐procedure directives was observed. At each visit, 7–11% of the participants tested positive for the presence of PSA compared to 19–25% during Phase I (Table 1). Overall, this represents a significant increase in the proportion of women adhering to the abstinence directive (*P* = 0.013). In Phase II, 18 of 62 participants (29%) tested PSA positive for at least one study point, compared to ten of sixteen (63%) in Phase I. When the participants were asked with whom they had unprotected sex during the post‐procedure period of abstinence, the answer given ranged from admitting having sex with a client or a regular partner, to denying having any sexual intercourse. Importantly, follow‐up HIV serology showed that none of the participants, in either phase of the study, seroconverted by 6 months after the end of the study.

During Phase II, those who decided to enroll in the study presented a higher level of understanding of the study risks and implications of the study than we observed in Phase I. We believe this contributed to the higher level of abstinence compliance observed in Phase II. Additionally, witnessing the detection of PSA (semen) in CVL at the point of care acted as an incentive to abstain from sex when required. Lack of compliance resulted in increased counseling and potential exclusion from the study if the nurse estimated that the participant was unlikely to respect the safety directives of the study. Overall, three participants were excluded from the study for their unwillingness to respect the post‐procedure directives following their enrollment. This PSA kit was well adapted for use in low‐to‐middle income settings with the advantage of being inexpensive, easy to use without special equipment requirement, or need for special training. Both clinical staff and the participants positively received the implementation of the PSA testing at the point of care. This was a clear advantage, as self‐declaration proved to be unreliable in Phase I. The study participants reported that the use of text messaging helped them remember to remain abstinent during the healing period. The participants and clinic staff reported that SMS and on‐site PSA testing were the methods that had the most impact on compliance to the post‐abstinence period.

Compliance was lower in our study than in Hasselrot's study,^7^ where they observed 97% of self‐reported and PSA tested compliance among FSWs and low‐risk women 3 days post‐procedure. In Hasselrot's study, participants were asked to abstain from sexual activities for at least 24 hr prior to the biopsy. This was not required in our study, and it is possible that sexual activity prior to the biopsy explains some of the weak positive PSA test we observed at visit 1, prior to the first biopsy sampling. The duration of the abstinence period was also longer in our study, reaching a full month. We observed the lowest level of compliance at the last visit (4), just before exiting this study (Table 2).

**Table 2:** Baseline Characteristics of Prostate‐Specific Antigen‐Positive and Prostate‐Specific Antigen‐Negative Female Sex Workers During Phase I and Phase II of the Study

Demographic and Behavioral Characteristics	Phase I		Phase II	
	PSA−		PSA−	
	(*n* = 6)	PSA+	(*n* = 44)	PSA+
	Median (IQR)	(*n* = 10)	Median (IQR)	(*n* = 18)
Age, in years	38 (36–44)	38 (35–41)	34 (31–39)	36 (30–40)
Duration of sex work, years	8 (7–10)	8 (5–12)	5 (2–10)	6 (3–10)
Number of clients in the last 7 days	18 (6–30)	7 (5–14)	5 (2–8)	6 (3–8)
Reported frequency of condom use with clients^&^	100 (100–100)	100 (87.5–100)	100 (100–100)	100 (100–100)
Reported frequency of condom use with the regular partner^&^	0 (0–100)	0 (0)	0 (0)	0 (0–100)
Schooling years	9 (7–10)	8 (7–14)	10 (8–12)	12 (10–13)**
Number of pregnancies	4 (3–6)	2 (2–3)*	2 (2–4)	3 (1–3)
	% (*n*)		% (*n*)	
Regular partner	33% (2)	70% (7)	57% (25)	78% (14)
Living with a man	0%	0%	7% (3)	11% (2)
Report consuming alcohol	0%	60% (6)#	71% (31)	72% (13)
Sexually transmitted infections (chlamydia or gonorrhea)	0%	0%	2% (1)	0%
Syphilis	0%	0%	0%	0%
HIV positive	33% (2)	50% (5)	5% (2)	28% (5)#

Analysis was performed in the women who completed the 4 study visits. *P < 0.05 between PSA− and PSA+, Mann–Whitney *U*‐test, **P < 0.01, ^#^ *P* < 0.05 between PSA− and PSA, Fischer's Exact Test, and Frequency of condom use for each participant were calculated as follows : number of sexual intercourses reporting using condom/number of sexual intercourses in the last 7 days × 100.

### Factors Associated with the Lack of Compliance

It is possible that factors such as ability to negotiate condom use, marital status, age, level of education, and experience in sex work may affect the participants' capability to comply with the post‐biopsy abstinence. However, in our study, age, duration of sex work, number of clients at baseline, number of pregnancies, having a regular partner, condom use, and marital status were not different between participants testing PSA positive for at least one visit and participants being PSA negative for all visits (Table 2). Interestingly, in Phase I, women who were positive for at least one PSA test reported consuming alcohol more often and had a reduced number of previous pregnancies than women testing negative for all PSA tests (Table 2). In Phase II, HIV‐infected women were disproportionally represented among the women testing positive for at least one PSA test (Table 2). Even if not statistically significant, the same trend was also observed in Phase I. Even though we did not collect any information on why HIV‐infected women failed to comply with the abstinence period, we can speculate that HIV‐positive women may have not perceived the risk of HIV infection the same as their HIV‐negative counterparts. It is also possible that HIV‐infected participants were more likely to adopt risky behaviors or have a decreased ability to negotiate abstinence with their partners.

### Procedure Outcomes

Healing was assessed for all participants through visual exam of the biopsy sites (Table 3) 3–5 days and again 14 days post‐biopsy. Signs of macroscopic hyperaemia, defined as any one of redness or presence of blood or swelling around the biopsy sites, were noticeable in most of the participants (72% and 75% of HIV‐negative and 40% and 46% of the HIV‐positive participants) 3–5 days post‐procedure, but had disappeared 14 days after the biopsy for the majority of the participants (97%, 60/62 for HIV negative and 100% (14/14) for HIV positive) (Table 3). As HIV infection is known to impair the immune response and result in chronic inflammation, the results are presented separately for HIV‐infected and HIV‐uninfected women. The two women who still showed sign of macroscopic hyperaemia during the visual inspection at the 14‐day follow‐up visit were advised not to resume sex work for at least one more week. Both women were HIV negative and had practiced sex work for more than 7 years. In the well‐defined cohort of Kenyan FSWs from Nairobi, these women are included within the HIV‐exposed sero‐negative (HESN) FSWs subset who are studied for their relative resistance to HIV acquisition conferred by a phenotype of immune quiescence.^12^ Wound healing triggers the recruitment and activation of granulocytes and monocytes at the site of biopsy healing, and the immune quiescence phenotype associated with HESN FSWs may explain why we observed decay in tissue repair within these two women. A previous report in the same cohort also observed that incomplete healing was more common within HESN participants when compared to non‐FSWs.^7^ Among HIV‐positive women, all demonstrated no signs of macroscopic hyperaemia at the time of exiting the study (Table 3). Being infected by HIV did thus not delay the healing process in general.

**Table 3:** Visual Inspection of Hyperaemia in HIV‐Uninfected and HIV‐Infected Female Sex Workers from Nairobi, Kenya. The Results from Phase I and Phase II were Merged

	HIV−			HIV+		
	PSA−	PSA+	Overall^b^	PSA−	PSA+	Overall°
Visit 1 (Biopsy 1)	2% (1/44)	11% (2/18)	5% (3/62)	10% (1/10)	0% (0/4)	7% (1/14)
Visit 2 (3–5 days post‐biopsy)	75% (47/63)	40% (2/5)	72% (49/68)***	46% (5/11)	25% (1/4)	40% (6/15)
Visit 3 (Biopsy 2)	7% (4/55)	17% (1/6)	8% (5/61)	23% (3/13)	0% (0/1)	21% (3/14)
Visit 4 (3–5 days post‐ biopsy)	78% (43/55)	50% (4/8)	75% (47/63)***	50% (5/10)	33% (1/3)	46% (6/13)*
Visit 5 (Final follow‐up)	4% (2/55)	0% (0/5)	3% (2/60)	0% (0/10)	0% (0/4)	0% (0/14)
*P* value^a^			<0.0001			0.0153

^a^Chi‐square to compare all visits, *P < 0.05, **P < 0.001, ***P < 0.0001 by Fishers Exact test when compared to visit 1.

^b^Based on the number of tests available, invalid tests were excluded.

**Figure 3:**
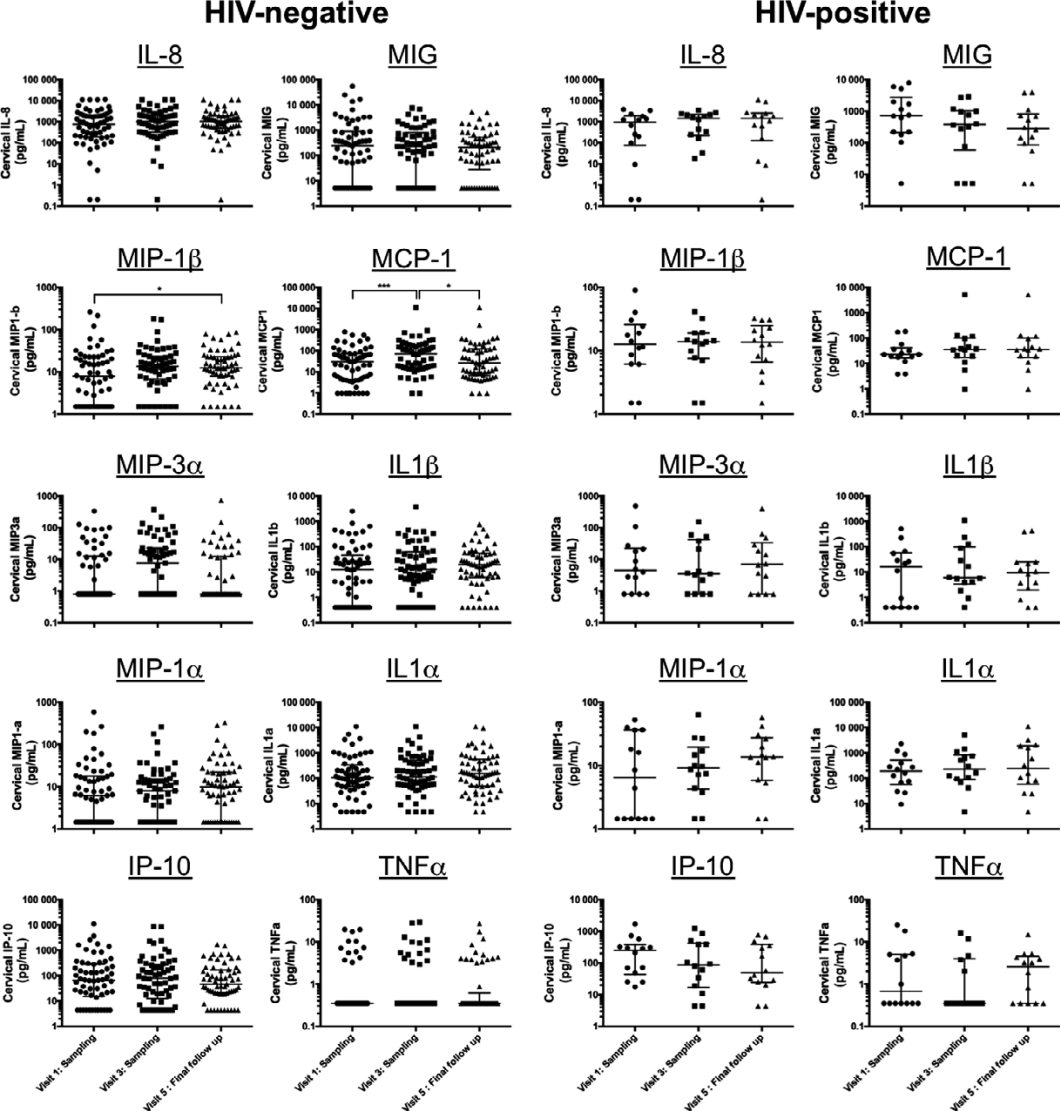
Concentration of pro‐inflammatory mediators in HIV‐uninfected and HIV‐infected FSW before and after biopsy sampling. Cervical concentrations of IL‐8, MIP1β, MIP3α, MIP1α, MCP‐1, MIG, IP‐10, IL‐1α, IL‐1β, and TNF‐α were measured by Milliplex (Millipore, Merck KGaA) in the cervicovaginal lavage collected at visit 1 (sampling), visit 3 (sampling), and visit 5 (final follow‐up). The difference in the cervical concentrations of inflammatory mediators at different visits was calculated using the nonparametric Friedman test for repeated measures. When significant, posthoc Dunn's multiple comparisons test was applied to compare between visits.

**Figure 4:**
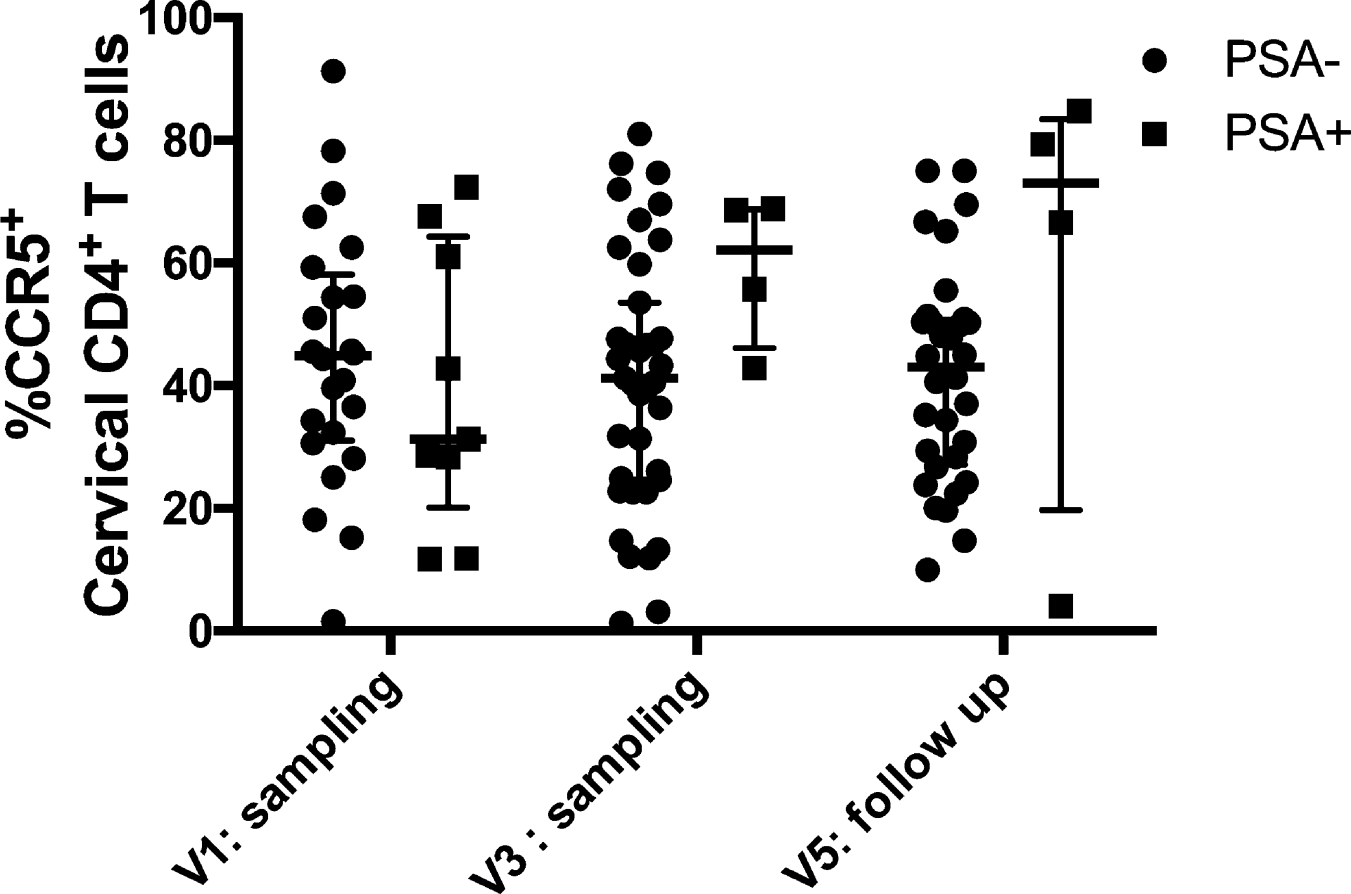
Proportion of cervical HIV T‐cell targets (CD4^+^CCR5^+^ T cells) in HIV‐uninfected and HIV‐infected FSWs before and after biopsy sampling. Cervical mononuclear cells were collected by endocervical cytobrush and ectocervical spatula scraping in PBS at visit 1 (sampling), visit 3 (sampling), and visit 5 (final follow‐up). Cells were stained for live cell discrimination and identification of HIV T‐cell targets (CD3, CD4, CCR5) by flow cytometry. The difference in the proportion of HIV T‐cell targets at different visits was calculated using the ordinary two‐way anova and the Sidak's multiple comparisons test.

Next, we asked whether performing a biopsy sampling would exacerbate inflammation as defined by a panel of cytokines and recruitment of HIV T‐cell targets (CD4^+^CCR5^+^ T cells) in the cervical compartment. We measured the cervical concentrations of the following inflammatory markers, MIP‐1α, MIP‐1β, IP‐10, IL‐8, MCP‐1, IL‐1α, IL‐1β, TNF‐α, MIG, and MIP‐3α, in the CVL of both HIV‐infected and HIV‐uninfected FSWs and the proportion of CD4^+^CCR5^+^ T cells in the cytobrush just before sampling and 14 days after the first and second biopsy sampling. Elevated cervical inflammation has been defined as an important catalyzer of HIV acquisition. Women who seroconverted during the CAPRISA 004 trial in South Africa had 5 of 9 pro‐inflammatory markers (MIP‐1α, MIP‐1β, IP‐10, IL‐8, MCP‐1, IL‐1α, IL‐1β, IL‐6, and TNF‐α) measured in the CVL being above the 75th percentile.^8^ We selected our panel of inflammatory makers according to Masson et al.^8^ and also included MIG and MIP3α as previous work from our team demonstrated that these markers are altered in the CVL of FSWs.^9^, ^10^ In the HIV‐sero‐negative women, eight of ten inflammatory mediators measured in CVL remained constant throughout all the sampling time points with MIP1β actually decreasing in the cervical compartment 14 days post‐sampling at study exit. The mean cervical concentrations of MCP‐1 increased 14 days after the procedure, but had returned to baseline levels when exiting the study (Fig. 3). We compared the proportion of women who showed signs of cervical inflammation (defined as having at least 5 of the 10 pro‐inflammatory markers above the 75th percentile) between visits. The proportion of women having an inflamed cervical environment did not significantly change between visits within HIV‐infected and HIV‐uninfected FSWs or according to the PSA status (Table 4). In line with these observations, the mean proportion of CCR5‐expressing CD4^+^ T cells among cervical T cells remained unchanged at all time points (Fig. 4), independent of the HIV and PSA status. Taken together, these results indicate that the 31 days of sexual abstinence were sufficient to allow proper healing of both biopsies and that biopsy sampling did not increase macroscopic hyperaemia, cervical concentration of a panel of pro‐inflammatory cytokines, or the number of HIV target cells in the FGT at study exit.

**Table 4:** Proportion of HIV‐Uninfected and HIV‐Infected Female Sex Workers from Nairobi, Kenya, with Cervical Inflammation, Defined as at Least 5/10 CVL Concentrations of Pro‐inflammatory Cytokines above the 75th Percentile. The Results from Phase I and Phase II were Merged

	HIV−			HIV+		
	PSA−	PSA+	Overall^a^	PSA−	PSA+	Overall^a^
Visit 1 (Biopsy 1)	16% (7/44)	11% (2/18)	15% (9/62)	20% (2/10)	25% (1/4)	21% (3/14)
Visit 3 (Biopsy 2)	9% (5/56)	33% (2/6)	11% (7/62)	15% (2/13)	0% (0/1)	14% (2/14)
Visit 5 (Final follow‐up)	15% (8/55)	0% (0/5)	13% (8/60)	10% (1/10)	25% (1/4)	14% (2/14)
*P* value^a^			0.8662			0.8425

Based on the number of tests available, invalid tests were excluded.

a Chi‐square to compare overall HIV uninfected and HIV infected for all visits.

### Limitations

We observed a discrepancy between the self‐reported sexual activity and positivity to PSA tests. Previous studies also reported a lack of reliability of self‐reported sexual activity,^13^, ^14^ indicating that studies only relying on self‐reported information may underestimate the sexual activity among their participants. While PSA testing does not allow for detection of protected intercourse, when considering the risk of HIV or other STIs infection, protected intercourse would be less harmful than unprotected intercourse.

## Conclusion

The study of the mucosal immune response is a priority in the field of HIV research, and understanding this distinct environment will be pivotal for the development of an efficient vaccine or microbicide. The traditional cervicovaginal sampling such as cytobrushes and CVL offer a convenient noninvasive alternative to explore the FGT immune response, but do not provide an opportunity to study the submucosal and intraepithelial milieu. Cervical biopsy sampling, on the other hand, offers a unique opportunity to understand the immune response present at the submucosal level. However, when performed in high‐risk populations, such as FSWs, concern can be raised about risk of HIV acquisition. By implementing three simple strategies, we improved the adherence to the post‐procedure abstinence by two times, limiting the risk of HIV acquisition following the procedure in a most‐at‐risk population of FSWs. We showed that PSA rapid testing at the point of care is an important procedure to encourage compliance, as self‐declaration was not always reliable. The use of PSA rapid testing allows for the provision of more immediate and personalized counseling. The use of bi‐weekly SMS offered an efficient method to remind the participants of the importance of not having sexual intercourse during the healing period. In addition, the comprehension consent tool improved understanding of the study requirements. Cervical biopsies, collected for research purpose, in high‐risk population are well tolerated and accepted in a well‐designed study, and 1 month of sexual abstinence is feasible among FSWs. Finally, 14 days after the biopsy sampling, no increase in the number of HIV T‐cell target or inflammatory response, as defined by a panel of selected cytokines in the cervical compartment could be observed. Cervical inflammation can induce the recruitment of HIV T‐cell target and is known to enhance the risk of acquiring HIV.^8^ Healing was completed after the 31 days of sexual abstention, and the genital environment was not affected by this procedure. Developing safe methods for studying the FGT environment will be critical for investigating the mucosal immune environment, which is crucial for designing effective interventions that prevent HIV infection.
